# From entrepreneurial learning engagement to entrepreneurial intention among Chinese university students: the roles of entrepreneurial self-efficacy and uncertainty avoidance

**DOI:** 10.3389/fpsyg.2026.1851960

**Published:** 2026-10-14

**Authors:** Pengzhong Dong, Chia Ching Tu, Xinmin Li, Limeng Dong

**Affiliations:** 1 Continuing Education College, Jilin Engineering Normal University, Changchun, Jilin, China; 2 Department of Educational Administration, International College, Krirk University, Bangkok, Thailand; 3 School of Data Science and Artificial Intelligence, Jilin Engineering Normal University, Changchun, Jilin, China; 4 Shenzhen Finance Institute, The Chinese University of Hong Kong, Shenzhen, Guangdong, China

**Keywords:** Chinese university students, entrepreneurial intention, entrepreneurial learning engagement, entrepreneurial self-efficacy, uncertainty avoidance

## Abstract

**Background:**

As universities play a growing role in fostering innovation and entrepreneurship to support economic growth, researchers have increasingly focused on understanding the mechanisms through which entrepreneurial education influences students' entrepreneurial intention. Accordingly, on the basis of learning engagement theory, social cognitive theory, and cultural dimension theory, this study investigated the relationships between entrepreneurial learning engagement, entrepreneurial self-efficacy, uncertainty avoidance, and entrepreneurial intention.

**Methods:**

Data were collected through a structured questionnaire administered to 1,578 university students in Jilin Province, China. Structural equation modeling (SEM) was employed to examine the direct, mediating, and moderating effects among the study variables.

**Results:**

Entrepreneurial learning engagement was positively associated with entrepreneurial intention both directly and indirectly through entrepreneurial self-efficacy. Multigroup analyses showed that its associations with self-efficacy and intention differed significantly between uncertainty-avoidance groups. In the high-avoidance group, only the indirect association was significant, whereas both direct and indirect associations were significant in the low-avoidance group. However, the between-group difference in the indirect association did not reach statistical significance.

**Conclusions:**

The findings highlight entrepreneurial self-efficacy as an important psychological correlate of both entrepreneurial learning engagement and entrepreneurial intention and indicate that these relationships differ across groups characterized by different levels of uncertainty avoidance. These patterns underscore the relevance of efficacy beliefs and uncertainty-related orientations for understanding students' entrepreneurial intention. Given the cross-sectional design, the findings should be interpreted as statistical associations rather than evidence of temporal ordering or causality. Future longitudinal or experimental research is needed to examine these relationships further.

## 1 Introduction

From a developmental psychology perspective, university students are at a pivotal stage of identity formation and career exploration, characterized by a growing need for autonomy, achievement motivation, and a future-oriented willingness to take risks (Arnett, 2000). This developmental period, commonly referred to as emerging adulthood, is associated with a greater tendency to pursue self-realization and construct social role identities through innovative activities and career experimentation (Obschonka et al., 2017). In response to these developmental characteristics, higher education systems worldwide have increasingly recognized entrepreneurship education as a strategic means of supporting students' innovative capabilities and employability [Organization for Economic Co-operation and Development (OECD), 2021].

Within the broader literature on higher education and entrepreneurship education, learning engagement is regarded as an important psychological resource associated with students' learning outcomes and subsequent behavioral intentions. It represents a positive and persistent learning-related psychological state characterized by vigor, dedication, and absorption (Schaufeli et al., 2002). By incorporating entrepreneurship-related curricula, promoting practice-oriented learning, and developing campus-based entrepreneurial ecosystems, universities seek to encourage students' active engagement in entrepreneurial learning. Previous studies have reported that participation in entrepreneurship courses, projects, and experiential learning activities is associated with greater entrepreneurship-related knowledge and skills (Wong and Chan, 2023) and stronger entrepreneurial intention (Liu et al., 2025; Sun et al., 2023; Wu et al., 2022). Accordingly, examining the relationship between entrepreneurial learning engagement and university students' entrepreneurial intention has important theoretical and practical implications.

The relationship between entrepreneurial learning engagement and entrepreneurial intention may involve both direct and indirect associations. According to social cognitive theory (Bandura, 1997), self-efficacy is an important psychological factor linking learning processes with behavioral intentions. Previous empirical studies have identified an indirect association between entrepreneurial learning engagement and entrepreneurial intention through entrepreneurial self-efficacy. Specifically, students with greater entrepreneurship-related knowledge, skills, and practical experience tend to report stronger confidence in their ability to start a business, which is associated with stronger entrepreneurial intention (Zhao et al., 2005; Vankov and Wang, 2024; Keshmiri, 2025; Newman et al., 2019). This pattern may be particularly relevant in Chinese higher education. Chinese university graduates face intense labor-market competition and employment challenges, within which entrepreneurship has become an increasingly salient career option (Wang and Verzat, 2011). In addition, Confucian traditions emphasize collectivism and family responsibility, suggesting that students' entrepreneurial decisions may reflect not only personal aspirations but also family and social expectations (Liñán and Chen, 2009). Accordingly, entrepreneurial self-efficacy may constitute an important psychological correlate within the observed relationship between entrepreneurial learning engagement and entrepreneurial intention among Chinese university students.

Uncertainty avoidance is a cultural orientation that may be relevant to the relationships described above. According to (Hofstede 2001a,b) cultural dimension theory, uncertainty avoidance reflects the extent to which individuals perceive ambiguous or unknown situations as threatening. Because entrepreneurship inherently involves considerable uncertainty, individual differences in uncertainty avoidance may correspond with variations in the relationship between entrepreneurial self-efficacy and entrepreneurial intention (Mueller and Thomas, 2001). Students with higher uncertainty avoidance may report weaker associations of learning engagement and self-efficacy with entrepreneurial intention, possibly reflecting greater concerns about failure and uncertain outcomes. By contrast, those with lower uncertainty avoidance may exhibit a closer association between learning engagement and entrepreneurial intention (Liñán and Fernandez-Serrano, 2014). In China, although traditional Confucian values emphasize stability and conformity, the promotion of the “Mass Entrepreneurship and Innovation” initiative and innovation-driven development strategies has coincided with increasingly diverse attitudes toward risk among university students (Fan et al., 2024; Huang et al., 2024; Lyu et al., 2024; Mensah et al., 2021). Accordingly, uncertainty avoidance may help account for heterogeneity in these relationships and provide a useful perspective for understanding how cultural orientations relate to students' entrepreneurial intentions.

This study examined the relationships among entrepreneurial learning engagement, entrepreneurial self-efficacy, and entrepreneurial intention among Chinese university students, as well as variations in these relationships across uncertainty-avoidance groups. By considering the indirect role of entrepreneurial self-efficacy and group differences associated with uncertainty avoidance, the study provides context-specific evidence regarding entrepreneurial intention within the distinctive labor-market and sociocultural context of Chinese higher education.

## 2 Literature review

### 2.1 Entrepreneurial learning engagement, entrepreneurial self-efficacy, and entrepreneurial intention among university students

International reports indicate that young people's interest in entrepreneurship remains strong amid increasing job insecurity and the growing consideration of entrepreneurship as a career option. However, a substantial gap persists between entrepreneurial intention and actual entrepreneurial entry, with limited financial resources, insufficient practical experience, and weak support systems frequently identified as relevant barriers (Dasgupta, 2022; Organization for Economic Co-operation and Development and European Commission, 2023). The Global Entrepreneurship Monitor (2024) also reported that entrepreneurship education remains inadequate in many countries, highlighting the importance of education and training in supporting the development of young people's entrepreneurial skills. From the perspective of social cognitive theory, efficacy beliefs are closely associated with individuals' action tendencies and represent an important cognitive factor in the relationship between learning experiences and behavioral intentions (Bandura, 1997).

Entrepreneurship education has expanded rapidly in Chinese universities in recent years, drawing increasing scholarly attention to university students' entrepreneurial learning engagement. Entrepreneurial learning engagement is commonly defined as the degree of cognitive, emotional, and behavioral involvement individuals demonstrate in entrepreneurship-related learning activities (Peterman and Kennedy, 2003). Greater engagement in such activities has been positively associated with entrepreneurial intention. Entrepreneurial intention refers to a psychological state in which individuals express a willingness to establish a new venture and formulate clear objectives and plans for doing so (Bird, 1988; Liñán and Fayolle, 2015; Thompson, 2009). Previous research has reported positive relationships among entrepreneurial learning engagement, opportunity-recognition capabilities, and entrepreneurial intention (Zhao et al., 2005). Entrepreneurial intention is also widely regarded as an important cognitive precursor to entrepreneurial behavior (Krueger, 2000).

Entrepreneurial self-efficacy may represent an important factor in the relationship between entrepreneurial learning engagement and entrepreneurial intention. It refers to individuals' confidence in their ability to perform entrepreneurial tasks successfully and has been associated with both entrepreneurship-related learning experiences and entrepreneurial intention (Chen et al., 1998). Individuals with stronger entrepreneurial self-efficacy also tend to report greater entrepreneurial intention and stronger entrepreneurial tendencies (Laviolette et al., 2012). Among Chinese university students, participation in entrepreneurship courses, practical training programs, competitions, and incubation activities may correspond with greater entrepreneurship-related knowledge, skills, mastery experiences, and self-efficacy. Entrepreneurial self-efficacy may therefore constitute a proximal psychological correlate within the indirect relationship between entrepreneurial learning engagement and entrepreneurial intention (Chen et al., 1998; Keshmiri, 2025; Vankov and Wang, 2024; Pan and Lu, 2022; Zhao et al., 2005). This theoretical relationship is also consistent with international youth development initiatives that emphasize supporting young people's skills and entrepreneurial capacities (United Nations, 2025). On the basis of the preceding discussion, this study proposed the following hypothesis:

1. H1: Entrepreneurial self-efficacy mediates the relationship between entrepreneurial learning engagement and entrepreneurial intention among university students.

### 2.2 Uncertainty avoidance, entrepreneurial learning engagement, entrepreneurial self-efficacy, and entrepreneurial intention among university students

Young people worldwide face increasing job insecurity amid the continuing socioeconomic consequences of the pandemic and global economic instability, while entrepreneurship remains a major focus of national policies and higher education systems. International reports have identified limited skills, resources, and institutional support as major barriers to youth entrepreneurship, highlighting the need for more effective entrepreneurship education and support systems designed to reduce barriers to entrepreneurial entry (International Labor Organization, 2024; Organization for Economic Co-operation and Development and European Commission, 2023). Furthermore, the Global Entrepreneurship Monitor (2024) reported that entrepreneurship education remains inadequate in many countries, reflecting a shared international concern regarding the relationships among learning experiences, entrepreneurial capabilities, and entrepreneurial intention.

Learning engagement theory proposes that active engagement is associated with stronger perceptions of learning control and self-efficacy, as well as more positive action tendencies and behavioral intentions (Fredricks et al., 2004). Among university students, engagement in entrepreneurship courses, project-based activities, competitions, and incubation programs may correspond with greater opportunities to acquire relevant skills and mastery experiences, as well as stronger entrepreneurial self-efficacy and intention (Chen et al., 1998; Zhao et al., 2005). Uncertainty avoidance, as a relatively stable value orientation, may also be relevant to individuals' perceptions of risk and ambiguity. Individuals with higher uncertainty avoidance tend to report stronger preferences for rules and predictability, together with greater concerns about risk when considering entrepreneurial decisions (Hofstede et al., 2010).

Previous research has reported that uncertainty avoidance and risk avoidance are negatively associated with entrepreneurship-related attitudes and intentions (Baluku et al., 2021; Calza et al., 2020). As a cultural orientation, uncertainty avoidance may be relevant to variations in these relationships. Hofstede (1984) and the GLOBE study (House et al., 2004) observed that individuals with higher uncertainty avoidance tend to be less comfortable with risk and ambiguity and to prefer structured knowledge, clear standards, and a stronger sense of control when contemplating action. For these individuals, entrepreneurial self-efficacy may represent an important psychological correlate within the indirect relationship between learning engagement and entrepreneurial intention.

Students with lower uncertainty avoidance tend to report greater tolerance for ambiguity and risk, together with stronger orientations toward learning, exploration, and creativity through practice. These characteristics are broadly consistent with the Schumpeterian entrepreneurial profile (Schumpeter, 1983; Mueller and Thomas, 2001), which emphasizes innovation, initiative, and a change-oriented mindset, as well as the perception of uncertainty as an opportunity rather than a threat (Shane, 2003). Accordingly, among students with lower uncertainty avoidance, entrepreneurial learning engagement may be indirectly associated with entrepreneurial intention through entrepreneurial self-efficacy while also exhibiting a direct association with entrepreneurial intention.

Overall, among students with higher uncertainty avoidance, entrepreneurial learning engagement may be less closely associated with self-efficacy, while concerns about failure and heightened risk perceptions may correspond with lower entrepreneurial intention. By contrast, students with lower uncertainty avoidance tend to demonstrate greater tolerance for ambiguity and risk, and their learning engagement may be more closely associated with entrepreneurial self-efficacy and intention. These contrasting patterns suggest that the strengths of the proposed relationships may vary across uncertainty-avoidance levels. In China, the institutionalization of innovation and entrepreneurship education following the introduction of the “Mass Entrepreneurship and Innovation” initiative in 2014 has coincided with the broader availability of entrepreneurial learning opportunities for university students. Nevertheless, individual differences in uncertainty avoidance may still correspond with heterogeneity in the associations among learning engagement, self-efficacy, and entrepreneurial intention. Accordingly, a multigroup mediation framework was considered appropriate for examining whether these associations differ between students with higher and lower uncertainty avoidance (Liu et al., 2017; Wang et al., 2022). On the basis of the preceding discussion, this study proposed the following hypothesis:

1. **H2:** The mediation model linking entrepreneurial learning engagement, entrepreneurial self-efficacy, and entrepreneurial intention differs across university students with different levels of uncertainty avoidance.

Figure 1 presents a conceptual model developed on the basis of H1 and H2.

**Figure 1:**
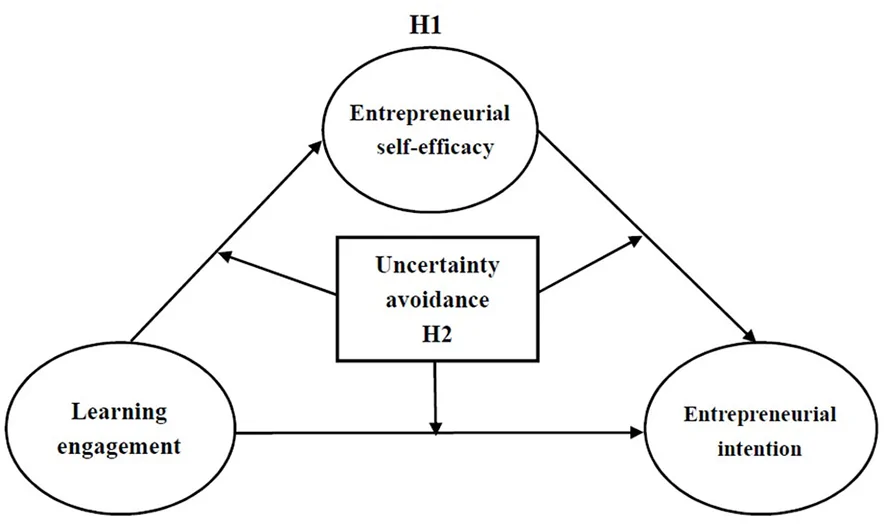
Conceptual model.

## 3 Methodology

### 3.1 Participants

All data collected in this study were used solely for research purposes and were collected and processed anonymously. Participants were informed of the purpose of the study, the voluntary nature of their participation, and their right to withdraw from the study. The research protocol was reviewed and approved by the Academic Committee of the International College, Krirk University, Thailand (Ref. No. 2022-096; approval date: December 22, 2022). The committee determined that the study posed no harm to the participants. All procedures were conducted in accordance with the ethical guidelines and requirements specified in the approved research protocol.

Purposive sampling was used to recruit students from seven universities in Jilin Province, China. Participants represented a wide range of academic disciplines, including science and engineering, economics and management, arts and sports, humanities, philosophy, law and education, and agriculture, forestry, and medical sciences. Across the seven participating universities, entrepreneurship education was generally delivered through a theoretical course titled Fundamentals of Entrepreneurship for first-year students, a 1-week entrepreneurship practicum for second-year students, and entrepreneurship competitions for third- and fourth-year students.

Questionnaires were distributed via email to 1,650 students in March 2023. A total of 1,578 valid questionnaires were returned, yielding an effective response rate of 95.6%. The final sample comprised 684 male students (43.35%) and 894 female students (56.65%). In terms of academic year, 481 participants were first-year students (30.5%), 543 were second-year students (34.4%), 361 were third-year students (22.9%), and 193 were fourth-year students (12.2%). Additional demographic characteristics of the participants are presented in Table 1.

**Table 1:** Demographic characteristics of the study sample.

Population variables	Categories	Frequency	Percentage
Gender	Male	684	43.35%
	Female	894	56.65%
Grade	Freshman	481	30.48%
	Sophomore	543	34.41%
	Third-year	361	22.88%
	Fourth-year	193	12.23%
Major	Science and Engineering	626	39.67%
	Economics and Management	459	29.09%
	Arts and Sports	146	9.25%
	Humanities, Philosophy, Law and Education	237	15.02%
	Agriculture, Forestry, and Medical Sciences	110	6.97%
Birth Place	City	671	42.52%
	Countryside	907	57.48%
Only child (binary: yes/no)	yes	778	49.3%
	no	800	50.7%
Joined innovation competition (binary: yes/no)	yes	525	33.27%
	no	1053	66.73%
Entrepreneurship experience (binary: yes/no)	yes	531	33.65%
	no	1047	66.35%
Family business background (binary: yes/no)	yes	642	40.68%
	no	936	59.32%

Source: Compiled by the researchers.

### 3.2 Research instruments

This study adopted a quantitative research approach involving a cross-sectional questionnaire survey design to examine the mechanisms through which entrepreneurial learning engagement influences entrepreneurial intention; the study focused on the mediating role of entrepreneurial self-efficacy and the moderating role of uncertainty avoidance. Structural equation modeling (SEM) was used to analyze the data and test the proposed hypotheses. Specifically, first, SPSS was used to conduct descriptive statistical and correlational analyses to examine the basic characteristics of the variables and their interrelationships. Subsequently, AMOS was used to conduct SEM analyses to test the hypotheses. Model fit was assessed using commonly accepted goodness-of-fit indices, including the comparative fit index (CFI), Tucker–Lewis index (TLI), and root mean square error of approximation (RMSEA), with reference to methodological discussions of model-fit evaluation (Mulaik et al., 1989; Browne and Cudeck, 1993; Hu and Bentler, 1999).

On the basis of established theories and the literature, this study conceptualized entrepreneurial learning engagement, entrepreneurial self-efficacy, and entrepreneurial intention as interrelated constructs. These three variables were measured using 5-point Likert-type scales with endpoints ranging from 1 (*strongly disagree*) to 5 (*strongly agree*).

(a) Entrepreneurial learning engagement refers to university students' cognitive, emotional, and behavioral involvement in entrepreneurship-related learning activities. This study adapted the 17-item student version of the Utrecht Work Engagement Scale (UWES; Schaufeli and Bakker, 2003). During the adaptation process, references to general studying and academic work were contextualized to reflect entrepreneurship-related learning activities, while the original conceptual meanings and three-factor structure of the items were retained. The adapted scale comprises three dimensions: vigor, dedication, and absorption.
(b) Entrepreneurial intention refers to university students' psychological readiness and willingness to engage in entrepreneurial activities. This study adopted the entrepreneurial intention scale developed by Yan and Ye (2009). This scale comprises seven items covering two dimensions: entrepreneurial likelihood and entrepreneurial behavioral tendency.
(c) Entrepreneurial self-efficacy (ESE) refers to individuals' perceived confidence in their ability to successfully perform various entrepreneurial tasks and activities. This study adopted a multidimensional scale grounded in the self-efficacy theory of Bandura (1997) and informed by prior research (Tang (2009). The scale comprises 22 items covering five dimensions: creative self-efficacy, risk-taking, opportunity recognition, relationship coordination, and organizational commitment.
(d) Uncertainty avoidance is a cultural value orientation that refers to the extent to which individuals respond to ambiguous and uncertain situations in manners that help them effectively avoid uncertainties (Veiga et al., 2001). This study adopted the uncertainty avoidance scale developed by Jung and Kellaris (2004). This scale is unidimensional and comprises seven items.

### 3.3 Measurement validity

Separate confirmatory factor analyses (CFAs) were conducted to evaluate the measurement models of entrepreneurial learning engagement, entrepreneurial self-efficacy, entrepreneurial intention, and uncertainty avoidance (Table 2). The Mardia coefficients for these four scales were 281.600, 461.474, 41.701, and 51.169, respectively, all of which were below their corresponding *p (p* + *2)* reference values of 323, 528, 63, and 63. These results indicated no substantial violation of multivariate normality (Bollen, 1989). In addition, all error variances were positive, and the standardized factor loadings ranged from 0.700 to 0.938, with none exceeding 1, indicating no apparent improper solutions.

**Table 2:** CFA model fit summary for the scales of entrepreneurial learning engagement, entrepreneurial self-efficacy, entrepreneurial intention, and uncertainty avoidance.

Variable	Entrepreneurial learning engagement	Entrepreneurial self-efficacy	Entrepreneurial intention	Uncertainty avoidance
Facets / items	3/17	5/22	2/7	1/7
*M*	3.100–3.228	3.368–3.476	2.905–3.014	2.923
*SD*	0.984–1.018	0.872–0.907	0.960–0.984	0.971
Cronbach's α	0.946–0.951	0.898–0.951	0.866–0.927	0.935
Standardized factor loadings	0.700–0.929	0.784–0.938	0.778–0.921	0.742–0.880
CR	0.946–0.954	0.898–0.951	0.869–0.928	0.936
AVE	0.759–0.778	0.688–0.829	0.721–0.769	0.677
Key fit indices	*χ^2^/df* = 20.172; GFI = 0.869; TLI = 0.914; CFI = 0.927; RMSEA = 0.110	*χ^2^/df* = 10.031; GFI = 0.886; TLI = 0.941; CFI = 0.949; RMSEA = 0.076	*χ^2^/df* = 10.918; GFI = 0.974; TLI = 0.974; CFI = 0.984; RMSEA = 0.079	*χ^2^/df* = 8.435; GFI = 0.978; TLI = 0.982; CFI = 0.988; RMSEA = 0.069

For multifaceted constructs, *M, SD*, Cronbach's α, CR, and AVE are presented at the facet level. Uncertainty avoidance is a single-factor construct. TLI is also known as NNFI. *M* = mean; *SD* = standard deviation; Cronbach's α = internal consistency reliability coefficient; CR = composite reliability; AVE = average variance extracted.

The incremental fit indices were generally acceptable, with NNFI (TLI) values ranging from 0.914 to 0.982 and CFI values ranging from 0.927 to 0.988. The RMSEA values for entrepreneurial self-efficacy (0.076), entrepreneurial intention (0.079), and uncertainty avoidance (0.069) were below 0.080, indicating acceptable approximate fit. In contrast, the entrepreneurial learning engagement model exhibited comparatively weaker fit, as reflected by its GFI of 0.869 and RMSEA of 0.110. Furthermore, the χ^2^/df ratios for the four models ranged from 8.435 to 20.172, exceeding commonly recommended thresholds. Although chi-square-based indices are sensitive to sample size, particularly in a large sample such as the present one (*N* = 1,578), the fit of the entrepreneurial learning engagement measurement model should be interpreted with caution (Bentler and Bonett, 1980; Marsh and Hocevar, 1985; Marsh et al., 1988).

Across the 11 facets represented by the four constructs, Cronbach's α coefficients ranged from 0.866 to 0.951, composite reliability (CR) values ranged from 0.869 to 0.954, and average variance extracted (AVE) values ranged from 0.677 to 0.829. These findings supported satisfactory internal consistency reliability and convergent validity. In addition, the square roots of the AVE ranged from 0.823 to 0.910 and exceeded the corresponding absolute inter-facet correlations, which ranged from 0.085 to 0.739, there by satisfying the Fornell and Larcker (1981) criterion.

Discriminant validity was further assessed using the heterotrait–monotrait ratio of correlations (HTMT). The HTMT values across the 11 facets ranged from 0.074 to 0.739, with the highest value observed between dedication and absorption (HTMT = 0.739). All values were below both the conservative threshold of 0.85 and the more liberal threshold of 0.90, providing additional evidence of discriminant validity (Henseler et al., 2015). Overall, the results supported the internal consistency reliability, convergent validity, and discriminant validity of the measures. Nevertheless, the comparatively weak fit of the entrepreneurial learning engagement measurement model should be considered when interpreting the subsequent structural model results.

### 3.4 Common method variance

Both ex ante procedural remedies and ex post statistical assessment were employed to address potential common method bias. During questionnaire administration, participants were informed that the survey was conducted solely for academic purposes, that their responses would remain confidential, and that the questionnaire was anonymous. These procedures were intended to reduce evaluation apprehension and socially desirable responding. Harman's single-factor test was subsequently conducted as an ex post diagnostic assessment. All measurement items were entered into an unrotated exploratory factor analysis, which yielded 10 factors with eigenvalues greater than 1. The first unrotated factor accounted for 35.436% of the total variance, below the 40% threshold adopted in this study (Podsakoff et al., 2003). Thus, common method bias did not appear to represent a serious threat to the interpretation of the findings.

## 4 Results and analysis

### 4.1 Structural model fit and mediation analysis

Bootstrapping was used to examine the indirect effect of entrepreneurial learning engagement on entrepreneurial intention through entrepreneurial self-efficacy. As shown in Figure 2, entrepreneurial learning engagement was positively associated with entrepreneurial self-efficacy (β = 0.523, *p* < 0.001), which, in turn, was positively associated with entrepreneurial intention (β = 0.592, *p* < 0.001). The model explained 27% of the variance in entrepreneurial self-efficacy and 42% of the variance in entrepreneurial intention (*R*^2^ = 0.27 and 0.42, respectively). The standardized indirect effect was approximately 0.310. The unstandardized indirect effect was statistically significant [*B*= 0.219, 95% CI (0.176, 0.276)], as the confidence interval excluded zero. The direct association also remained statistically significant [β = 0.102, *p* < 0.01; *B* = 0.072, 95% CI (0.018, 0.127)]. Taken together, these findings supported the partial mediating role of entrepreneurial self-efficacy in the association between entrepreneurial learning engagement and entrepreneurial intention, thereby supporting H1 (Figure 2 and Table 3).

**Figure 2:**
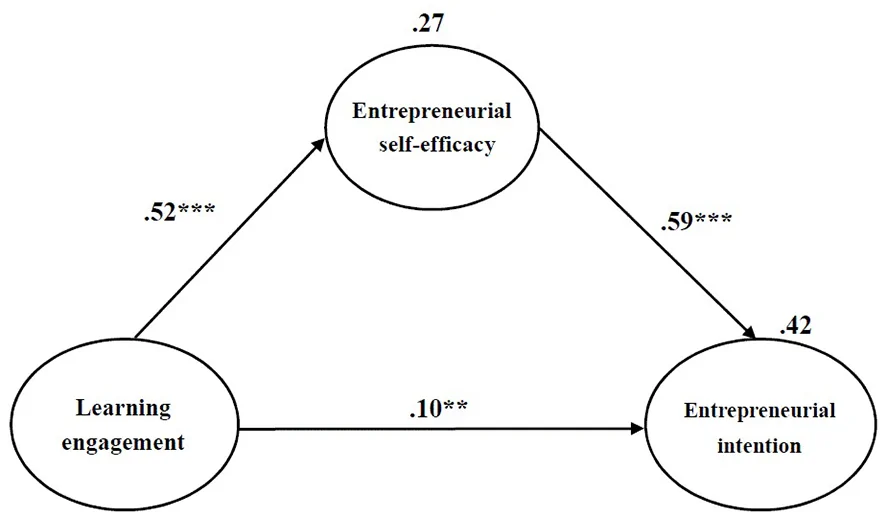
Mediation model for the full sample. ^**^Indicates *p* < 0.01, and ^***^indicates *p* < 0.001.

**Table 3:** Bootstrap estimates of the unstandardized effects of entrepreneurial learning engagement on entrepreneurial intention.

Mediating effect	*B*	95% Confidence interval	
		Lower bound	Upper bound
Total group			
Total effect	0.291^***^	0.234	0.354
Direct effect	0.072^**^	0.018	0.127
Indirect effect	0.219^***^	0.176	0.276
High uncertainty avoidance group			
Total effect	0.134	0.000	0.237
Direct effect	−0.022	−0.149	0.072
Indirect effect	0.156^***^	0.093	0.240
Low uncertainty avoidance group			
Total effect	0.374^***^	0.297	0.460
Direct effect	0.119^***^	0.038	0.195
Indirect effect	0.255^***^	0.194	0.334

Unstandardized effects are reported; ^**^ *p* < 0.01; ^***^p < 0.001.

### 4.2 Moderating effect of uncertainty avoidance

#### 4.2.1 K-means clustering and external validation of uncertainty-avoidance groups

Although uncertainty avoidance is a continuous psychological characteristic, *K*-means clustering was conducted using its seven items to facilitate comparisons of structural relationships between students with different levels of uncertainty avoidance. By minimizing within-cluster variation, this procedure generated relatively homogeneous groups (Chen, 2010; Hair et al., 2019). The 1,578 participants were classified into a low-uncertainty-avoidance group (*n* = 1,094) and a high-uncertainty-avoidance group (*n* = 484). Their mean uncertainty avoidance scores were 2.374 and 4.164, respectively, supporting the designation of the two groups.

To assess the theoretical interpretability of the clustering solution, the groups were compared on theoretically relevant variables that were not included in the clustering procedure. The low-uncertainty-avoidance group reported significantly higher entrepreneurial self-efficacy (*M* = 3.524, SD = 0.666) than the high-uncertainty-avoidance group (M = 3.215, SD = 0.842), Welch's *t* (761.84) = 7.149, *p* < 0.001, Cohen's *d* = 0.43. The low-uncertainty-avoidance group also reported higher entrepreneurial intention (*M* = 2.995, SD = 0.854) than the high-uncertainty-avoidance group (M = 2.880, SD = 0.842), *t* (1,576) = 2.465, *p* = 0.014, *d* = 0.14. Similarly, entrepreneurial learning engagement was higher in the low-uncertainty-avoidance group (*M* = 3.220, SD = 0.852) than in the high-uncertainty-avoidance group (*M* = 2.975, SD = 0.892), *t* (1,576) = 5.188, *p* < 0.001, *d* = 0.28. According to Cohen (1988), the corresponding effect sizes were small to moderate, very small, and small, respectively. Overall, these findings provided **supplementary** external evidence for the theoretical interpretability of the clustering solution.

#### 4.2.2 Measurement invariance testing

Prior to the multigroup structural model analysis, measurement invariance across the high–and low-uncertainty-avoidance groups was assessed within the mean and covariance structures (MACS) framework (Little, 1997). Following Cheung and Rensvold (2002) and Chen (2007), a stepwise nested-model comparison procedure was employed, beginning with the configural model (M1) and proceeding to the metric model (M2).

The configural model demonstrated an acceptable fit, χ^2^ (64) = 405.009, χ^2^/df = 6.328, CFI = 0.955, TLI = 0.937, RMSEA = 0.058, and SRMR = 0.0341, supporting the same latent factor structure across the two groups. Metric invariance was subsequently examined by constraining the corresponding factor loadings to equality across groups. The metric model also demonstrated an acceptable fit, χ^2^ (71) = 426.597, χ^2^/df = 6.008, CFI = 0.953, TLI = 0.941, RMSEA = 0.056, and SRMR = 0.0355.

Relative to the configural model, changes in the approximate fit indices were minimal: ΔCFI = −0.002, ΔRMSEA = −0.002, and ΔSRMR = 0.0014. These changes remained well within the recommended thresholds, namely, an absolute decrease in CFI not exceeding 0.010, an increase in RMSEA not exceeding 0.015, and an increase in SRMR not exceeding 0.030 (Cheung and Rensvold, 2002; Chen, 2007). Although the chi-square difference test was statistically significant, Δχ^2^(7) = 21.588, *p* = 0.003, this statistic is sensitive to large sample sizes. Given the minimal changes in the approximate fit indices, imposing equality constraints on the factor loadings did not result in a substantive deterioration in model fit. Metric invariance was therefore supported, indicating that entrepreneurial learning engagement, entrepreneurial self-efficacy, and entrepreneurial intention were measured on a comparable metric across the two uncertainty-avoidance groups. This result supported subsequent multigroup comparisons of the structural path coefficients.

#### 4.2.3 Group differences in the structural model across levels of uncertainty avoidance

After metric invariance had been established, multigroup structural equation modeling was conducted to examine whether the structural paths in the mediation model differed between the low- and high-uncertainty-avoidance groups. An unconstrained model, in which all structural paths were freely estimated across groups, was first specified. This model was then compared with a constrained model in which the three paths—entrepreneurial learning engagement to entrepreneurial self-efficacy, entrepreneurial self-efficacy to entrepreneurial intention, and entrepreneurial learning engagement to entrepreneurial intention—were constrained to equality across groups.

The unconstrained model demonstrated an acceptable fit, χ^2^ (64) = 405.009, χ^2^/df = 6.328, AGFI = 0.916, NFI = 0.948, CFI = 0.955, and RFI = 0.926. The constrained model also showed an acceptable fit, χ^2^ (67) = 426.702, χ^2^*/df* = 6.369, AGFI = 0.915, NFI = 0.945, CFI = 0.953, and RFI = 0.926. Although the χ^2^*/df* ratios were relatively high, the remaining indices indicated an acceptable overall fit (Hair et al., 1998; Lomax and Schumacker, 2004). The chi-square difference test was statistically significant, Δχ^2^(3) = 21.693, *p* < 0.001, indicating that the structural paths were not fully equivalent across the two groups. Individual paths were therefore examined to identify the sources of these between-group differences (Paulssen et al., 2014; Rodríguez et al., 2008).

Figure 3 presents the standardized path coefficients and explained variances, whereas Table 4 reports the unstandardized coefficients and *Z* tests of between-group differences. The association between entrepreneurial learning engagement and entrepreneurial self-efficacy was stronger in the low-uncertainty-avoidance group (β = 0.61, *p* < 0.001; *B* = 0.452, *p* < 0.001) than in the high-uncertainty-avoidance group (β = 0.34, *p* < 0.001; *B* = 0.311, *p* < 0.001). The between-group difference was statistically significant (*Z* = −2.601, *p* = 0.009).

**Figure 3:**
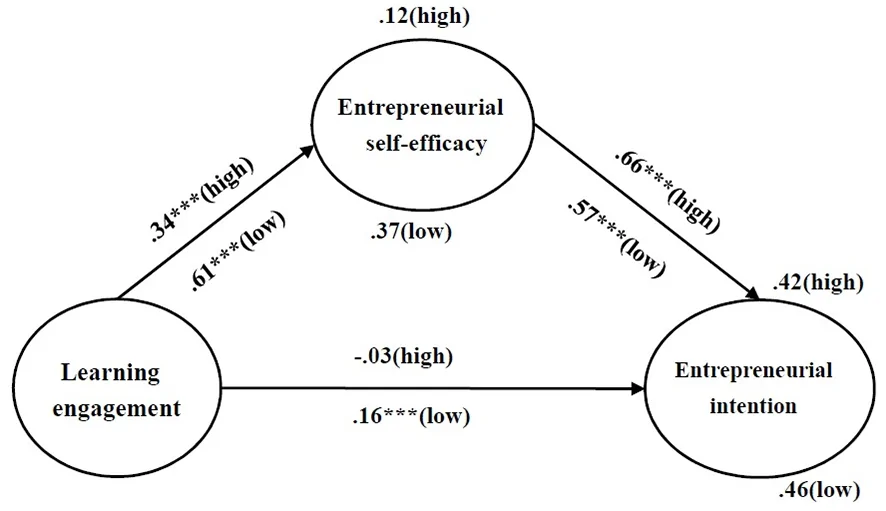
Mediating model for individuals with high and low levels of uncertainty avoidance. ^***^Indicates *p* < 0.001.

**Table 4:** Pairwise comparisons of structural path coefficients between the low–and high-uncertainty-avoidance groups.

Pathway	Low		High		*Z*
	*B*	*p*	B	*p*	
Number of participants	1,094		484		
Entrepreneurial learning engagement → entrepreneurial self-efficacy	0.452^***^	< 0.001	0.311^***^	< 0.001	−2.601^**^
Entrepreneurial learning engagement → entrepreneurial intention	0.119^***^	< 0.001	−0.022	0.582	−2.795^**^
Entrepreneurial self-efficacy → entrepreneurial intention	0.564^***^	< 0.001	0.501^***^	< 0.001	−0.713

*B* = *unstandardized path coefficient*. ^**^ *P* < 0.01; ^***^ *P* < 0.001; *Z* values were calculated by subtracting the estimate for the low-uncertainty-avoidance group from that for the high-uncertainty-avoidance group.

The direct association between entrepreneurial learning engagement and entrepreneurial intention was also stronger in the low-uncertainty-avoidance group. This path was positive and significant in the low-uncertainty-avoidance group (β = 0.16, *p* < 0.001; *B* = 0.119, *p* < 0.001), but non-significant in the high-uncertainty-avoidance group (β = −0.03; *B* = −0.022, *p* = 0.582). The between-group difference was statistically significant (*Z* = −2.795, *p* = 0.005).

Entrepreneurial self-efficacy was positively associated with entrepreneurial intention in both the low-uncertainty-avoidance group (β = 0.57, *p* < 0.001; *B* = 0.564, *p* < 0.001) and the high-uncertainty-avoidance group (β = 0.66, *p* < 0.001; *B* = 0.501, *p* < 0.001). However, the between-group difference was not statistically significant (Z = −0.713, *p* = 0.476). Thus, despite the numerical difference between the standardized coefficients, there was insufficient evidence of a significant group difference in this path.

As shown in Figure 3, the model explained 37% and 12% of the variance in entrepreneurial self-efficacy in the low- and high-uncertainty-avoidance groups, respectively (*R*^2^ = 0.37 and 0.12). It also explained 46% and 42% of the variance in entrepreneurial intention in the respective groups (*R*^2^ = 0.46 and 0.42). Thus, entrepreneurial learning engagement accounted for substantially more variance in entrepreneurial self-efficacy among students with lower uncertainty avoidance, whereas the model demonstrated relatively similar explanatory power for entrepreneurial intention across the two groups.

#### 4.2.4 Group-specific mediation and moderated mediation effects

Bootstrapping was conducted to examine the indirect effect within each uncertainty-avoidance group. As shown in Table 3, which reports unstandardized effects, the total effect [B = 0.374, 95% CI (0.297, 0.460)], direct effect [*B* = 0.119, 95% CI (0.038, 0.195)], and indirect effect through entrepreneurial self-efficacy [*B* = 0.255, 95% CI (0.194, 0.334)] were all statistically significant in the low-uncertainty-avoidance group. Because the confidence intervals for both the direct and indirect effects excluded zero, entrepreneurial self-efficacy partially mediated the association between entrepreneurial learning engagement and entrepreneurial intention in this group.

In the high-uncertainty-avoidance group, the indirect effect was statistically significant [*B* = 0.156, 95% CI (0.093, 0.240]), whereas the direct effect was not [*B* = −0.022, 95% CI (−0.149, 0.072)]. The total effect did not reach the conventional level of statistical significance [*B* = 0.134, 95% CI (0.000, 0.237), *p* = 0.051]. This pattern—comprising a significant indirect effect and a non-significant direct effect—was consistent with indirect-only mediation.

Based on the standardized path coefficients presented in Figure 3, the standardized indirect effects were approximately 0.348 (0.61 × 0.57) and 0.224 (0.34 × 0.66) in the low- and high-uncertainty-avoidance groups, respectively. When calculated by subtracting the estimate for the low-uncertainty-avoidance group from that for the high-uncertainty-avoidance group, the difference in the standardized indirect effects was −0.124 (0.224–0.348), with a bootstrapped 95% confidence interval of [−0.198, 0.000], *p* = 0.0501. Because the confidence interval included zero and the *p* value exceeded 0.05, this difference was not statistically significant. Thus, although the indirect effect was significant within each group and numerically larger in the low-uncertainty-avoidance group, its magnitude did not differ significantly across groups.

Overall, in the full sample, entrepreneurial learning engagement was significantly and positively associated with entrepreneurial intention, both directly and indirectly through entrepreneurial self-efficacy (Figure 2; Table 3). The multigroup analyses further revealed significant differences in the paths from entrepreneurial learning engagement to entrepreneurial self-efficacy and from entrepreneurial learning engagement to entrepreneurial intention, with both associations being stronger in the low-uncertainty-avoidance group (Figure 3; Table 4). However, neither the path from entrepreneurial self-efficacy to entrepreneurial intention nor the overall indirect effect differed significantly across groups. Thus, the findings supported the mediation hypothesis (H1) and provided partial support for H2: group differences were observed in two specific structural paths, but not in the remaining path or the overall indirect effect.

## 5 Discussion

This study examined the relationships among entrepreneurial learning engagement, entrepreneurial self-efficacy, and entrepreneurial intention among Chinese university students, as well as variations in these relationships across uncertainty-avoidance groups. The findings are broadly consistent with the theoretical perspectives discussed in the literature and provide context-specific evidence from Chinese higher education.

### 5.1 Relationship between entrepreneurial learning engagement and entrepreneurial intention

The results showed that entrepreneurial learning engagement was significantly and positively associated with entrepreneurial intention among university students. This finding is consistent with learning engagement theory, which emphasizes the relevance of students' cognitive, emotional, and behavioral involvement in learning activities (Fredricks et al., 2004). The observed association is also consistent with previous studies reporting positive relationships between participation in entrepreneurship courses, projects, and experiential activities and entrepreneurial intention (Liu et al., 2025; Sun et al., 2023; Wu et al., 2022). One possible theoretical interpretation is that students with greater entrepreneurial learning engagement may have more opportunities to acquire entrepreneurship-related knowledge and become familiar with opportunity recognition and problem-solving processes. These learning experiences may, in turn, be associated with stronger entrepreneurial intention.

### 5.2 The indirect role of entrepreneurial self-efficacy

The mediation analysis showed that entrepreneurial learning engagement was significantly associated with entrepreneurial intention both directly and indirectly through entrepreneurial self-efficacy, thereby supporting H1. This result is consistent with previous empirical studies reporting an indirect association between entrepreneurship-related learning experiences and entrepreneurial intention through entrepreneurial self-efficacy (Chen et al., 1998; Keshmiri, 2025; Vankov and Wang, 2024; Pan and Lu, 2022; Zhao et al., 2005). Students reporting greater entrepreneurial learning engagement also tended to report stronger confidence in their ability to perform entrepreneurial tasks, and such confidence was associated with stronger entrepreneurial intention. This pattern aligns with social cognitive theory, which identifies self-efficacy as an important cognitive correlate of behavioral choices (Bandura, 1997). The simultaneous presence of significant direct and indirect associations further suggests that entrepreneurial self-efficacy is an important, but not exclusive, psychological factor in the relationship between entrepreneurial learning engagement and entrepreneurial intention.

### 5.3 Differences in structural relationships across uncertainty-avoidance groups

The observed between-group differences were broadly consistent with Hofstede's cultural dimension theory, which associates lower uncertainty avoidance with greater tolerance for ambiguity and less reliance on predictability and formal structures (Hofstede, 1984; Hofstede et al., 2010; House et al., 2004). In the present study, entrepreneurial learning engagement was more strongly related to both entrepreneurial self-efficacy and entrepreneurial intention among students with lower uncertainty avoidance. This pattern is consistent with previous research linking tolerance for uncertainty to innovation-oriented entrepreneurial characteristics and stronger entrepreneurial intention (Mueller and Thomas, 2001; Liñán and Fernandez-Serrano, 2014). Accordingly, individual differences in uncertainty avoidance may be relevant not only to students' entrepreneurial intentions but also to how learning engagement relates to their entrepreneurial confidence and career considerations. Students with lower uncertainty avoidance may perceive entrepreneurship-related learning as more compatible with exploration and uncertain outcomes, whereas those with higher uncertainty avoidance may regard such learning as less compatible with their preference for predictability and structure. This interpretation is consistent with previous research indicating that risk avoidance is negatively associated with entrepreneurship-related attitudes and intentions (Baluku et al., 2021; Calza et al., 2020). However, because the present study did not directly assess risk tolerance, fear of failure, exploratory motivation, or perceptions of ambiguity, these factors should be regarded as plausible theoretical explanations rather than empirically established mechanisms.

In the high-uncertainty-avoidance group, the significant indirect effect, coupled with the non-significant direct effect, indicated an indirect-only mediation pattern. This finding suggests that entrepreneurial learning engagement was related to entrepreneurial intention primarily through entrepreneurial self-efficacy. From a social cognitive perspective, learning experiences may be linked to behavioral intentions through perceptions of competence and personal control (Bandura, 1997). Entrepreneurial self-efficacy may therefore represent a potential explanatory link between learning engagement and entrepreneurial intention among students with higher uncertainty avoidance. Nevertheless, because perceptions of control and psychological safety were not directly measured, this interpretation should be treated with caution. By contrast, the low-uncertainty-avoidance group exhibited a partial mediation pattern. Entrepreneurial learning engagement was positively related to entrepreneurial self-efficacy, which was, in turn, positively related to entrepreneurial intention, while the direct relationship between learning engagement and entrepreneurial intention remained significant. This pattern is broadly consistent with the Schumpeterian entrepreneurial profile, which emphasizes innovation, initiative, and the pursuit of emerging opportunities (Schumpeter, 1983; Mueller and Thomas, 2001). Students with lower uncertainty avoidance may be more receptive to entrepreneurship-related learning experiences that encourage exploration, creativity, and autonomous decision-making. Such experiences may therefore be related to entrepreneurial intention both directly and indirectly through enhanced self-efficacy (Krueger and Brazeal, 1994; Shane, 2003). Because these motivational and interpretive processes were not directly measured, however, they should be regarded as tentative explanations.

Contrary to expectations derived from cultural dimension theory, the relationship between entrepreneurial self-efficacy and entrepreneurial intention did not differ significantly between the two uncertainty-avoidance groups. This finding is more consistent with social cognitive theory, which conceptualizes self-efficacy as a proximal, task-specific cognition relevant to behavioral choice (Bandura, 1997). It also accords with previous research demonstrating a robust positive relationship between entrepreneurial self-efficacy and entrepreneurial intention (Chen et al., 1998; Newman et al., 2019; Zhao et al., 2005). As a domain-specific belief, entrepreneurial self-efficacy may be more closely related to entrepreneurial intention than the broader orientation of uncertainty avoidance. The participants' shared educational and sociocultural context in Jilin Province may also have contributed to the similarity of this relationship across the two groups. Taken together, the findings support the theoretical relevance of individual uncertainty-avoidance orientations to entrepreneurial cognition and intention. At the individual level, tolerance for uncertainty may be associated with how entrepreneurial learning experiences relate to self-efficacy and entrepreneurial intention (Liñán and Chen, 2009; Rauch and Frese, 2007). Students with lower uncertainty avoidance may be more receptive to innovation- and action-oriented learning, whereas those with higher uncertainty avoidance may place greater value on structured learning and the development of competence beliefs. These patterns highlight the potential importance of considering individual differences in uncertainty avoidance when designing entrepreneurship education.

Overall, uncertainty avoidance was associated with differences in some, but not all, of the hypothesized structural relationships. Specifically, the relationships of entrepreneurial learning engagement with entrepreneurial self-efficacy and entrepreneurial intention differed significantly between the two groups, whereas the relationship between entrepreneurial self-efficacy and entrepreneurial intention did not. Although the low-uncertainty-avoidance group exhibited partial mediation and the high-uncertainty-avoidance group exhibited indirect-only mediation, the between-group difference in the overall indirect effect through entrepreneurial self-efficacy was not statistically significant. These findings provide partial support for H2 and indicate that the observed heterogeneity was concentrated in specific structural paths rather than in the overall indirect effect.

From a practical perspective, the findings suggest that entrepreneurship education may benefit from considering differences in students' uncertainty-avoidance orientations. Students with higher uncertainty avoidance may benefit from clearer learning structures, progressive goals, risk-assessment training, and predictable feedback intended to strengthen their entrepreneurial capability beliefs (Bandura, 1997; Hofstede et al., 2010). By contrast, students with lower uncertainty avoidance may benefit from greater opportunities for open-ended exploration, innovation-oriented experimentation, and autonomous decision-making (Mueller and Thomas, 2001; Vankov and Wang, 2024). Nevertheless, these recommendations represent theoretically informed implications rather than interventions directly evaluated in the present study. Their effectiveness should therefore be examined in future longitudinal or intervention-based research.

## 6 Theoretical and practical implications

### 6.1 Theoretical implications

This study integrates entrepreneurial learning engagement, entrepreneurial self-efficacy, entrepreneurial intention, and uncertainty avoidance within a framework encompassing learning engagement, efficacy beliefs, and cultural orientations. Unlike prior studies examining these factors separately (De Jorge-Moreno et al., 2012; Ismail et al., 2015; Liñán and Fayolle, 2015; Piperopoulos and Dimov, 2015), it considers their direct and indirect associations and between-group differences, offering an integrated theoretical perspective on entrepreneurial intention.

First, the findings are consistent with learning engagement theory, which highlights the relevance of cognitive, emotional, and behavioral involvement (Fredricks et al., 2004), and with social cognitive theory, which emphasizes efficacy beliefs in the cognition–intention relationship (Bandura, 1997). In entrepreneurial learning contexts, these perspectives suggest that learning engagement alone may not fully account for entrepreneurial intention; entrepreneurial capability beliefs are important correlates (Newman et al., 2019; Piperopoulos and Dimov, 2015).

Second, this study extends prior average-association research by showing that relationships involving entrepreneurial learning engagement vary across student groups according to orientations toward ambiguity and risk (Liñán and Fayolle, 2015; Usaci, 2015). By examining uncertainty avoidance as an individual-level orientation in entrepreneurial psychology, the study moves beyond its macro-level treatment, aligns with cultural dimension theory (Hofstede, 1984, 2001a,b; Hofstede et al., 2010), and highlights its relevance to structural heterogeneity.

Finally, group differences were observed in paths linking entrepreneurial learning engagement to entrepreneurial self-efficacy and entrepreneurial intention, whereas the overall indirect association did not differ significantly between groups. Accordingly, uncertainty avoidance may be viewed as an orientation associated with path-strength differences rather than a boundary condition for the overall indirect association. Distinguishing local path heterogeneity from overall moderated mediation clarifies the theoretical role of cultural orientations in these relationships.

### 6.2 Practical implications

This study offers several practical implications for universities and entrepreneurship education institutions. First, the positive associations among entrepreneurial learning engagement, entrepreneurial self-efficacy, and entrepreneurial intention suggest that entrepreneurship programs may place greater emphasis on experiential, practice-oriented, and project-based learning. Such activities are theoretically relevant to students' perceived control, mastery, and beliefs in their entrepreneurial capabilities (Kuh, 2001a,b; Kuh et al., 2008).

Second, the observed differences between the uncertainty-avoidance groups suggest the potential value of differentiated instruction. Programs for students with high uncertainty avoidance may incorporate risk-management content, structured guidance, and opportunities to reflect on failure and ambiguity. These approaches are consistent with the psychological orientations associated with uncertainty avoidance (Hayton et al., 2002; Lemke and Baum, 2021; Nguyen et al., 2009).

Third, universities may consider offering diversified support systems, including psychological counseling, mentoring, and peer coaching. These resources may provide students with opportunities to discuss entrepreneurial uncertainty and strengthen their perceived preparedness for entrepreneurial activities. Nevertheless, because the present findings are cross-sectional, the effectiveness of these educational strategies should be evaluated through longitudinal or intervention-based research.

## 7 Conclusions

Overall, the findings highlight the joint relevance of entrepreneurial learning engagement, entrepreneurial self-efficacy, and uncertainty-related orientations for understanding university students' entrepreneurial intention. Entrepreneurial self-efficacy represented an important psychological correlate within the indirect association between learning engagement and entrepreneurial intention, while specific structural relationships differed between the high- and low-uncertainty-avoidance groups. Nevertheless, the between-group difference in the overall indirect association was not statistically significant. These findings suggest that efficacy beliefs and uncertainty avoidance offer complementary perspectives on variation in entrepreneurial intention. Given the cross-sectional design, the observed relationships should be interpreted as associations rather than causal effects.

### 7.1 Limitations and future directions

This study has several limitations. First, its cross-sectional design precludes conclusions regarding temporal ordering or causality, and reverse or reciprocal relationships remain possible. Future longitudinal or experimental studies are therefore needed. Second, reliance on self-report data may have introduced social desirability and shared-method variance; future research could incorporate qualitative or multisource data. Third, although the sample was substantial, participants were purposively recruited from seven universities in Jilin Province, limiting the generalizability of the findings. More geographically and institutionally diverse samples are recommended. Finally, classifying continuous uncertainty avoidance indicators into high- and low-level groups through K-means clustering may have resulted in information loss. Future studies could complement multigroup comparisons with continuous moderation or latent interaction analysis.

## Data Availability

The datasets presented in this study can be found in online repositories. The names of the repository/repositories and accession number(s) can be found at: The datasets generated and/or analyzed during the current study are available in the Open Science Framework (OSF) repository, accessible at: https://osf.io/9pn42?view_only=725d9d3ed6cd44ff805199891f3a7689. All data are openly shared with view-only access for the research community.
