# Supplementary material for: From entrepreneurial learning engagement to entrepreneurial intention among Chinese university students: the roles of entrepreneurial self-efficacy and uncertainty avoidance
Source: Front Psychol. 2026 Oct 14;17:1851960. doi: 10.3389/fpsyg.2026.1851960 (PMC13713649; doi:10.3389/fpsyg.2026.1851960)
Supplement: Supplementary file 1 [file Data_Sheet_1.pdf]

Supplementary Material

Supplementary Tables 1–7 provide a comprehensive assessment of the psychometric properties of the four constructs examined in this study: Entrepreneurial Learning Engagement, Entrepreneurial Self-Efficacy, Entrepreneurial Intention, and Uncertainty Avoidance. Table 1 presents the fit indices for the four measurement models. Tables 2–5 report item-level descriptive statistics and standardized factor loadings, together with facet-level internal consistency reliability, composite reliability, and average variance extracted. Tables 6 and 7 present the correlations and assessments of discriminant validity among the eleven facets. As shown in Table 6, the square root of the AVE for each facet exceeds its correlations with the other facets. In addition, all HTMT values reported in Table 7 are below .85. Taken together, these results provide support for the discriminant validity of the measurement model.

TABLE 1

Model Fit Indices for the Measurement Models of the Four Variables

Statistical test index		Criterion	Entrepreneurial learning engagement	Entrepreneurial self-efficacy	Entrepreneurial intention	Uncertainty avoidance
Absolute fit indices	χ^2	Smaller is better	2340.008	1966.252	141.930	118.090
	χ^2/df	<3	20.172	10.031	10.918	8.435
	GFI	>.900	.869	.886	.974	.978
	RMSEA	<.080	.110	.076	.079	.069
Incremental fit indices	NNFI	>.900	.914	.941	.974	.982
	CFI	>.900	.927	.949	.984	.988
	RFI	>.900	.910	.935	.972	.979
Parsimonious fit indices	PNFI	>.500	.788	.813	.608	.657
	PGFI	>.500	.659	.697	.452	.489

Note. χ^2 = chi-square statistic; χ^2/df = chi-square-to-degrees-of-freedom ratio; GFI = goodness-of-fit index; RMSEA = root mean square error of approximation; NNFI = non-normed fit index, also known as the Tucker–Lewis index (TLI); CFI = comparative fit index; RFI = relative fit index; PNFI = parsimonious normed fit index; PGFI = parsimonious goodness-of-fit index.

TABLE 2

Descriptive Statistics, Reliability, and Validity of the Entrepreneurial Intention Scale （N=1578）

Dimensions	Items	*SK*	*KU*	*SFL*	*EV*	*SE*	*M*	*SD*	Cronbach's α	CR	AVE
Entrepreneurial likelihood	yy1	-0.158	-0.192	.830	.328	.025	2.899	1.028	.866	.869	.769
	yy2	-0.184	-0.262	.921	.173	.030	3.129	1.068			
entrepreneurial behavioral tendency	yy3	-0.220	-0.634	.778	.492	.020	3.110	1.116	.927	.928	.721
	yy4	0.097	-0.398	.821	.363	.015	2.696	1.056			
	yy5	-0.017	-0.693	.840	.342	.015	2.835	1.079			
	yy6	-0.143	-0.648	.897	.237	.012	3.010	1.100			
	yy7	-0.019	-0.609	.904	.221	.011	2.876	1.101			
		Mardia=41.701		P*(P+2)=63							

Note. SK = skewness; KU = kurtosis; SFL = standardized factor loading; EV = error variance; SE = standard error; M = mean; SD = standard deviation; Cronbach’s α = internal consistency reliability coefficient; CR = composite reliability; AVE = average variance extracted.

TABLE3

Descriptive Statistics, Reliability, and Validity of the Entrepreneurial Learning Engagement Scale（N=1578）

Facets	Items	*SK*	*KU*	*SFL*	*EV*	*SE*	*M*	*SD*	Cronbach's α	CR	AVE
Vigor	tr1	0.103	-0.660	.813	.433	.017	2.854	1.132	.949	.950	.759
	tr2	-0.246	-0.632	.861	.322	.013	3.132	1.117			
	tr3	-0.169	-0.687	.842	.372	.015	3.226	1.130			
	tr4	-0.159	-0.526	.913	.203	.009	3.117	1.105			
	tr5	-0.216	-0.448	.913	.195	.009	3.143	1.080			
	tr6	-0.175	-0.433	.882	.254	.011	3.128	1.070			
Dedication	tr7	-0.256	-0.382	.844	.329	.014	3.212	1.071	.946	.946	.777
	tr8	-0.274	-0.648	.869	.309	.013	3.249	1.123			
	tr9	-0.219	-0.864	.901	.254	.012	3.203	1.163			
	tr10	-0.250	-0.715	.898	.247	.011	3.203	1.131			
	tr11	-0.291	-0.563	.895	.252	.011	3.274	1.126			
Absorption	tr12	-0.251	-0.805	.700	.684	.025	3.202	1.159	.951	.954	.778
	tr13	-0.192	-0.446	.927	.164	.007	3.103	1.081	.		
	tr14	-0.283	-0.410	.923	.171	.008	3.150	1.075			
	tr15	-0.204	-0.423	.929	.158	.007	3.105	1.074			

tr16	-0.078	-0.465	.911	.201	.009	3.008	1.085
tr17	-0.279	-0.598	.881	.279	.011	3.180	1.116
	Mardia=281.60		P*(P+2)=323				

Note. SK = skewness; KU = kurtosis; SFL = standardized factor loading; EV = error variance; SE = standard error; M = mean; SD = standard deviation; Cronbach’s α = internal consistency reliability coefficient; CR = composite reliability; AVE = average variance extracted.

TABLE 4

Descriptive Statistics, Reliability, and Validity of the of Entrepreneurial Self-Efficacy Scale （N=1578）

Facets	Items	*SK*	*KU*	*SFL*	*EV*	*SE*	*M*	*SD*	Cronbach's α	CR	AVE
Creative self-efficacy	xn1	-0.536	-0.100	.794	.396	.015	3.494	1.036	.947	.947	.750
	xn2	-0.542	0.041	.924	.146	.007	3.509	.995			
	xn3	-0.347	-0.151	.938	.121	.006	3.381	1.007			
	xn4	-0.556	-0.034	.852	.286	.012	3.549	1.022			
	xn5	-0.410	0.127	.830	.281	.011	3.409	.950			
	xn6	-0.405	0.100	.850	.256	.010	3.390	.959			
Risk-taking	xn7	-0.470	0.186	.835	.272	.013	3.452	.949	.898	.898	.688
	xn8	-0.338	-0.287	.784	.407	.017	3.290	1.028			
	xn9	-0.401	-0.259	.847	.302	.014	3.396	1.032			
	xn10	-0.298	-0.170	.852	.261	.013	3.335	.978			
Opportunity recognition	xn11	-0.462	0.116	.901	.177	.008	3.421	.971	.951	.951	.829
	xn12	-0.391	0.012	.921	.143	.007	3.433	.971			
	xn13	-0.364	-0.027	.918	.149	.007	3.397	.976			
	xn14	-0.414	0.125	.902	.174	.008	3.394	.966			
Relationship coordination	xn15	-0.445	-0.161	.850	.288	.013	3.438	1.107	.927	.926	.759
	xn16	-0.357	0.033	.862	.236	.011	3.392	.958			
	xn17	-0.436	-0.174	.876	.237	.011	3.479	1.010			
	xn18	-0.393	-0.295	.897	.205	.010	3.433	1.023			
Organizational commitment	xn19	-0.455	0.124	.909	.156	.008	3.507	.950	.940	.941	.801
	xn20	-0.551	0.213	.934	.114	.006	3.556	.947			
	xn21	-0.258	-0.546	.894	.213	.010	3.414	1.029			
	xn22	-0.307	-0.409	.842	.293	.012	3.428	1.003			
	Mardia=461.474		P*(P+2)=528								

Note. SK = skewness; KU = kurtosis; SFL = standardized factor loading; EV = error variance; SE = standard error; M = mean; SD = standard deviation; Cronbach’s α = internal consistency reliability coefficient; CR = composite reliability; AVE = average variance extracted.

TABLE 5

Descriptive Statistics, Reliability, and Validity of the Uncertainty Avoidance Scale （N=1578）

Facet	Items	*SK*	*KU*	*SFL*	*EV*	*SE*	*M*	*SD*	Cronbach's α	CR	AVE
Uncertainty avoidance	un1	0.130	-0.903	.859	.343	.015	2.926	1.145	.935	.936	.677
	un2	0.038	-0.780	.860	.320	.014	2.978	1.108			
	un3	0.082	-0.716	.880	.270	.012	2.959	1.096			
	un4	0.026	-0.817	.800	.506	.020	2.871	1.186			
	un5	0.081	-0.807	.753	.573	.022	2.843	1.152			
	un6	-0.005	-0.944	.742	.635	.024	2.885	1.188			
	un7	-0.047	-0.759	.857	.339	.015	2.997	1.130			
	Mardia=51.169		P*(P+2)=63								

Note. SK = skewness; KU = kurtosis; SFL = standardized factor loading; EV = error variance; SE = standard error; M = mean; SD = standard deviation; Cronbach's α = internal consistency reliability coefficient; CR = composite reliability; AVE = average variance extracted.

TABLE 6

Correlations and Discriminant Validity

Facets	Number of items	Correlation Coefficients										
		A	B	C	D	E	F	G	H	I	J	K
A.Vigor	6	.871										
B.Dedication	5	.634***	.881									
C.Absorption	6	.652***	.675***	.882								
D.Creative self-efficacy	6	.335***	.384***	.326***	.866							
E.Risk-taking	4	.306***	.315***	.270***	.654***	.829						
F.Opportunity recognition	4	.284***	.397***	.317***	.603***	.674***	.910					
G.Relationship coordination.	4	.265***	.385***	.302***	.657***	.630***	.666***	.871				
H.Organizational commitment	4	.374***	.449***	.367***	.620***	.572***	.599***	.739***	.895			
I.Entrepreneurial likelihood	2	.147***	.165***	.140***	.389***	.361***	.394***	.328***	.289***	.877		
J.Entrepreneurial behavioral tendency	5	.333***	.346***	.307***	.515***	.455***	.504***	.436***	.414***	.583***	.849	
K.Uncertainty avoidance	7	-.181***	-.251***	-.125***	-.241***	-.199***	-.237***	-.238***	-.289***	-.085**	-.109***	.823

Note: 1. *p <0.05，**p <0.01，***p<0.001

2. The diagonal values represent the square roots of the average variance extracted (AVE).

TABLE 7

HTMT Matrix for the Eleven First-Order Facets

Facets	A	B	C	D	E	F	G	H	I	J	K
A.Vigor	—										
B.Dedication	0.638	—									
C.Absorption	0.674	0.739	—								
D.Creative self efficacy	0.328	0.375	0.346	—							
E.Risk taking	0.304	0.317	0.294	0.668	—						
F.Opportunity recognition	0.278	0.402	0.345	0.606	0.669	—					
G.Relationship coordination.	0.263	0.385	0.334	0.648	0.633	0.669	—				
H.Organizational commitment	0.378	0.452	0.405	0.626	0.577	0.606	0.738	—			
I.Entrepreneurial likelihood	0.137	0.161	0.144	0.400	0.363	0.390	0.330	0.288	—		
J.Entrepreneurial behavioral tendency	0.328	0.346	0.330	0.520	0.454	0.504	0.461	0.443	0.598	—	
K.Uncertainty avoidance	0.180	0.251	0.147	0.243	0.193	0.236	0.239	0.296	0.074	0.108	—

Note. HTMT = heterotrait–monotrait ratio of correlations. Values are reported below the diagonal. All HTMT values were below the conservative threshold of .85, providing support for discriminant validity.
